# Population-level normative models reveal race- and socioeconomic-related variability in cortical thickness of threat neurocircuitry

**DOI:** 10.1038/s42003-024-06436-7

**Published:** 2024-06-19

**Authors:** Nathaniel G. Harnett, Negar Fani, Grace Rowland, Poornima Kumar, Saige Rutherford, Lisa D. Nickerson

**Affiliations:** 1 Division of Depression and Anxiety, McLean Hospital, Belmont, MA USA; 2 Department of Psychiatry, Harvard Medical School, Boston, MA USA; 3 Department of Psychiatry and Behavioral Neuroscience, Emory University, Atlanta, GA USA; 4 McLean Imaging Center, McLean Hospital, Belmont, MA USA; 5 Department of Cognitive Neuroscience, Radboud University Nijmegen Medical Centre, Nijmegen, Netherlands; 6 Donders Institute, Radboud University Nijmegen, Nijmegen, Netherlands; 7 Department of Psychiatry, University of Michigan-Ann Arbor, Ann Arbor, MI USA; 8 Applied Neuroimaging Statistics Research Laboratory, McLean Hospital, Belmont, MA USA

**Keywords:** Neural circuits, Computational neuroscience, Scientific community

## Abstract

The inequitable distribution of economic resources and exposure to adversity between racial groups contributes to mental health disparities within the United States. Consideration of the potential neurodevelopmental consequences, however, has been limited particularly for neurocircuitry known to regulate the emotional response to threat. Characterizing the consequences of inequity on threat neurocircuitry is critical for robust and generalizable neurobiological models of psychiatric illness. Here we use data from the Adolescent Brain and Cognitive Development Study 4.0 release to investigate the contributions of individual and neighborhood-level economic resources and exposure to discrimination. We investigate the potential appearance of race-related differences using both standard methods and through population-level normative modeling. We show that, in a sample of white and Black adolescents, racial inequities in socioeconomic factors largely contribute to the appearance of race-related differences in cortical thickness of threat neurocircuitry. The race-related differences are preserved through the use of population-level models and such models also preserve associations between cortical thickness and specific socioeconomic factors. The present findings highlight that such socioeconomic inequities largely underlie race-related differences in brain morphology. The present findings provide important new insight for the generation of generalizable neurobiological models of psychiatric illness.

## Abstract (web summary)

A structural MRI study that suggests race-related differences in the cortical thickness of adolescents are partially driven by inequities in exposure to economic resources and discrimination.

## Introduction

Socioeconomic resources are inequitably distributed across groups within the United States. In general, Black individuals and communities have lower wealth and more neighborhood disadvantage compared to white individuals and communities^1,2^. Both individual access to, and spatial distribution of, economic resources influence opportunities for growth, health, and positive outcomes across the lifespan. Importantly, racially marginalized – particularly Black–individuals face greater exposure to racial discrimination at both the interpersonal and societal level throughout development^3,4^. Discrimination and socioeconomic inequity can contribute to disruptions in emotion regulation that is tied to the development of internalizing psychopathology such as posttraumatic stress disorder and depression in the future^5,6^. Further, each of the above factors are associated with alterations in neural structure of key brain regions (e.g., amygdala, hippocampus, and prefrontal cortex; PFC) known to regulate the emotional response to threat and that play a significant role in internalizing psychopathology^7–10^. Racial inequities may therefore contribute to race-related variability in neurodevelopment with potential downstream consequences for emotional function. However, research investigating the confluence of both socioeconomic deprivation and discrimination at both individual and society levels on the brain is nascent. Failure to appropriately contextualize racial inequities in developmental outcomes tied to economic resources risks limiting our understanding of neurodevelopment and lessening the efficacy of appropriate interventions for internalizing psychopathology.

Emergent research over the past five years has highlighted both individual and societal-level influences that may influence race-related variability in threat neurobiology. The extant literature highlights that variability in socioeconomic factors are robustly associated with brain morphology development^11,12^. For example, lower income and greater neighborhood poverty are associated with reduced volume of the amygdala, hippocampus, and PFC^13,14^. Prior work also highlights that cortical morphology is differentially associated with individual and neighborhood level metrics of poverty^15^. Importantly, racial inequity in these factors across adolescence largely accounts for race-related differences in neural reactivity to threat in young adults^16^. Interpersonal racial discrimination is also associated with alterations in threat-related brain regions. Racial discrimination is associated with enhanced connectivity of the amygdala to regions involved in attention to biologically salient stimuli such as the insula and PFC^17^. Further, discrimination is associated with greater activation within the ventromedial PFC, a key brain area involved in emotion regulation, in trauma-exposed Black women above and beyond symptoms of PTSD^10^. The same cohort further demonstrated reduced white matter microstructure of the dorsal cingulum and cortical thickness of the anterior cingulate cortex, key brain structures implicated in attention to threat^18,19^. Recent work utilizing the Adolescent Brain and Cognitive Development (ABCD) Study, a multisite longitudinal study of brain development in approximately 12,000 children, demonstrated state-level indices of racial bias (i.e., structural stigma) were associated with reduced volume of the hippocampus of Black children^4^. Further, the functional connectome of children in the ABCD study was associated with their socioeconomic status, which in turn mediated observed cognitive function^20^. Similarly, racial inequities in socioeconomic factors such as income, neighborhood disadvantage, and interpersonal dynamics like family conflict partially explain race-related differences in gray matter volume of threat-related brain regions of participants within the ABCD Study^21^. The extant literature thus highlights that both individual and structural factors impact the function and structure of threat neurocircuitry and such effects are observable in early development. However, limited work has investigated the combined influence of these metrics and if they may explain, in part, race-related variability in brain development.

Given the scale of racial inequity within the U.S., neuroimaging approaches that model typical patterns of, and deviations from, “typical” brain development may provide useful insights on the neurodevelopmental impact of racial inequity that are key to precision medicine. Population-based approaches in particular can identify robust brain-behavior associations relevant to clinical populations^22,23^. Further, mega-analyses of numerous large scale neuroimaging studies can be used to construct normative models of brain structure^24,25^. Normative modeling allows for quantification of an individual’s deviation from a population norm, and these deviations may provide more insight than standard approaches, such as case-control comparisons, for neuropsychiatric disorders such as schizophrenia^26,27^. Racial inequity and race-related stress may alter neurodevelopment and contribute to differences from the “general” population. However, the extent to which population-based modeling approaches may capture race- and socioeconomic-related variance is not well established. Prior work using normative modeling approaches have shown effects of traumatic stress on morphology when controlling for race-related effects^28^, but limited consideration has been given to the interplay of these factors on race-related differences. Evaluation of the sensitivity of population-level models to identify race- and socioeconomic-related effects on brain morphology may allow for a better understanding of the neurodevelopmental consequences of early life adversity across the human population.

The present study sought to address two unexplored areas relative to modeling of racial inequities in neurodevelopmental outcomes. We first sought to determine if race-related differences were present in cortical thickness of threat neurocircuitry. In line with a growing literature on the effects of race-related stressors, we hypothesized that (a) race-related differences would be present in cortical thickness, (b) individual (i.e., family income and discrimination) and neighborhood (i.e., neighborhood disadvantage and state-level racism) factors would be linked to cortical thickness, and (c) disparities in individual and neighborhood factors between racial groups would partially mediate race-related differences in cortical thickness. We then sought to investigate if population-based normative models may provide greater sensitivity to race and socioeconomic-related variability in brain structure. Thus, we hypothesized that a normative modeling approach would reveal race-related differences in cortical thickness that would be attenuated by disparities in the individual and neighborhood factors. The current findings provide new insight into the neurobiological consequences of racial disparities and the usage of population-based approaches for investigating sociodemographic variability in neurobiology.

## Results

Consistent with prior work, white children resided in significantly more advantaged neighborhoods and had caregivers with more income at the second year assessment compared to Black children (Table 1). Further, Black children experienced more racial discrimination and resided in areas with more state-level racism compared to white children. Though the groups did not differ in age, there was a difference in the proportion of children assigned male or female at birth with a more equal proportion observed in Black children recruited to the ABCD study compared to white children. Notably, the ABCD Study sites differed from each other in terms of average income [F(20,6029) = 48.11, *p* < 0.001, R^2^ = 0.14], ADI [F(20,6078) = 189.40, *p* < 0.001, R^2^ = 0.39], discrimination [F(20,6483) = 38,600, *p* < 0.001, R^2^ = 0.03], and state-level racism [F(20,6029) = 48.11, *p* < 0.001, R^2^ = 0.99].

**Table 1:** Participant demographics

	Total (*n* = 6505)	White (*n* = 5393)	Black (*n* = 1112)	Test statistic
Age	11.94 (0.64)	11.94 (0.64)	11.94 (0.64)	t (1602.6) = 0.05, *p* = 0.96
Sex assigned at birth				χ2 = 4.85, *p* = 0.028
Male	2967 (46%)	2426 (45%)	541 (49%)	
Female	3538 (54%)	2967 (55%)	571 (51%)	
Income				t (1116.8) = 31.63, *p* < 0.001
<$5,000	150 (2%)	47 (1%)	103 (9%)	
$5000–$11999	170 (3%)	56 (1%)	114 (10%)	
$12000–$15999	107 (2%)	58 (2%)	49 (4%)	
$16000–$24999	196 (3%)	121 (4%)	75 (7%)	
$25000–$34999	308 (5%)	192 (4%)	116 (10%)	
$35000–$49999	386 (6%)	269 (5%)	117 (11%)	
$50000–$74999	806 (12%)	638 (12%)	168 (15%)	
$75000–$99999	881 (14%)	799 (15%)	82 (7%)	
$100000–$199999	2177 (33%)	2073 (38%)	104 (9%)	
>$200000	869 (13%)	850 (16%)	19 (2%)	
Missing	455 (7%)	290 (5%)	165 (15%)	
Discrimination	8.23 (3.10)	7.92 (2.59)	9.88 (4.67)	t (1068.8) = 12.57, *p* < 0.001
Area Deprivation Index	40.01 (26.00)	34.86 (22.35)	66.33 (27.34)	t (1272.2) = 34.21, *p* < 0.001
State-level Racism	–0.18 (0.77)	–0.23 (0.78)	0.08 (0.68)	t (1764.9) = 13.73, *p* < 0.001

*T* tests are Welch two sample t-tests to adjust for inhomogeneity of variance between groups.

**Fig. 1:**
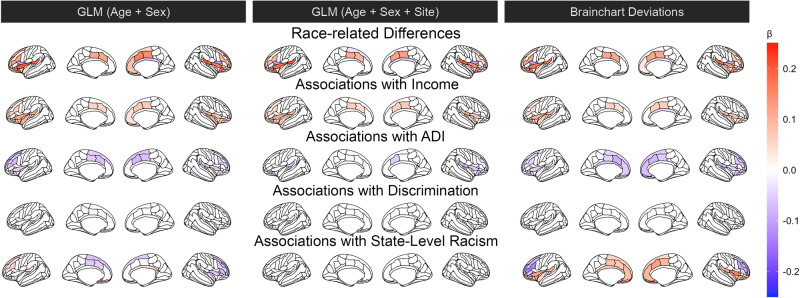
Race- and socioeconomic-related variation in cortical thickness of threat neurocircuitry. Linear models were used to assess race-related (top row) or socioeconomic-related (bottom four rows) variation in cortical thickness of threat neurocircuitry adjusting for age and sex (left column), age, sex, and site (middle column), or using deviations from normative models (right column). Colors reflect standardized Beta values from the regression analyses (calculated using lm.beta from the QuantPsyc package) for the strength of the effect. Only effects significant at q < 0.05 are shown. L = Left, R = Right.

**Fig. 2:**
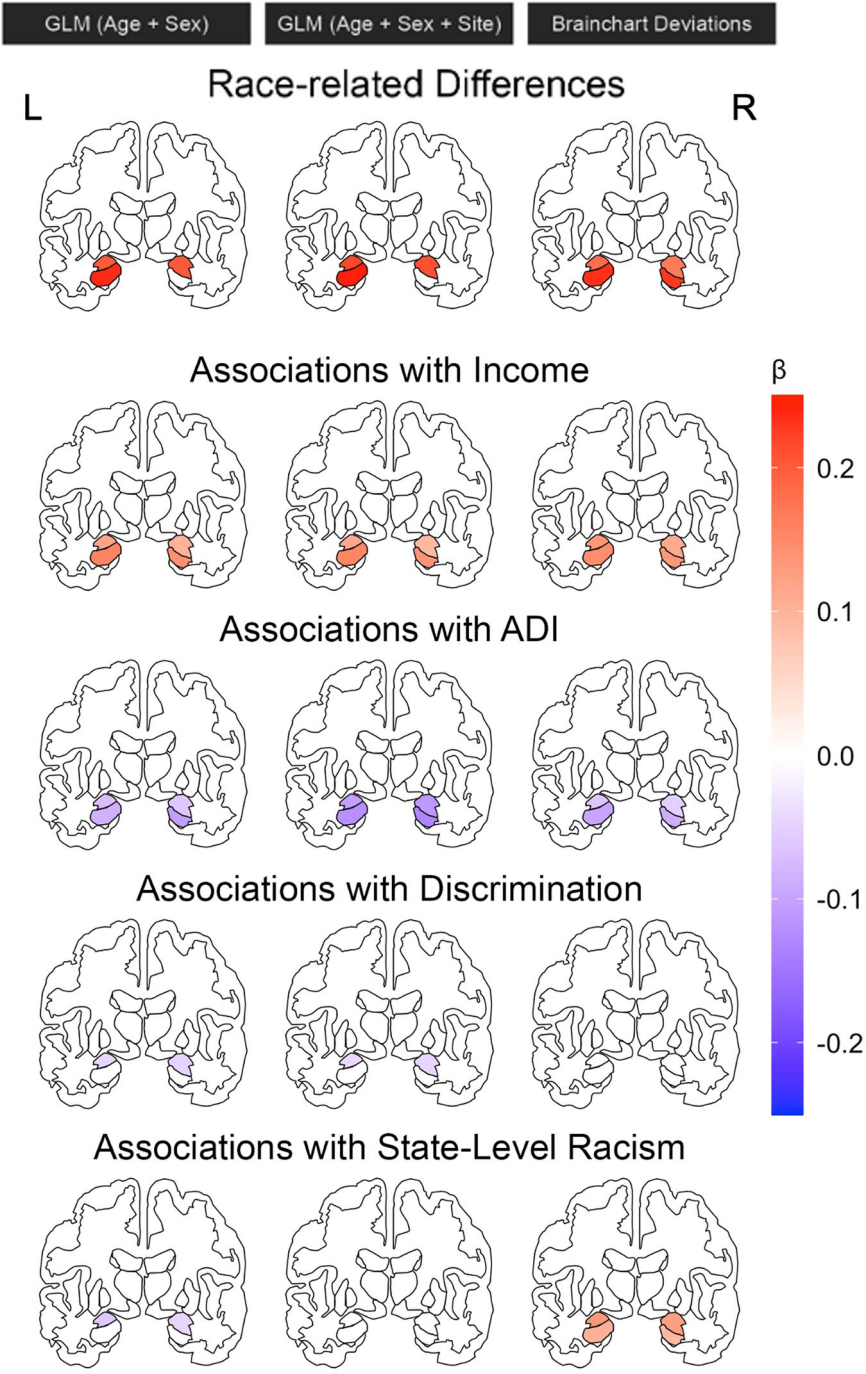
Race- and socioeconomic-related variation in amygdala-hippocampal volume. Linear models were used to assess race-related (top row) or socioeconomic-related (bottom four rows) variation in volume of the amygdala and hippocampus adjusting for age and sex (left column), age, sex, and site (middle column), or using deviations from normative models (right column). Colors reflect standardized Beta values from the regression analyses (calculated using lm.beta from the QuantPsyc package) for the strength of the effect. Only effects significant at q < 0.05 are shown. L = Left, R = Right.

We observed significant race-related differences in cortical thickness across many of the a priori regions of interest (Fig. 1; Table S2). We observed notable race-related differences in thickness of the cingulate and ventromedial PFC as well as volume of the hippocampus (Fig. 2). Inclusion of site as a covariate in the analyses somewhat modified the observed race-related differences across threat neurocircuitry. Race-related differences were also observed in the Z-score deviations derived from the normative model. In general, not accounting for site in standard linear models led to larger race-related effects that were slightly attenuated with site was included in the model. Further, utilizing the Z-score deviations preserved some effects (e.g., differences in anterior cingulate thickness) while also revealing unique effects (e.g., suborbital thickness). Thus, there are observable race-related differences in the morphology of threat neurocircuitry that are preserved in population-normed models.

Individual and neighborhood socioeconomic factors were also associated with cortical thickness of threat neurocircuitry (Figs. 1, 2). Income was positively associated with thickness of several brain regions even after accounting for site and with population-level normative models with fairly consistent effects across the models (Table S3). Similarly, ADI (Table S4) was associated with cortical thickness with and without accounting for site though more regions showed significant effects with the population-level normative models. In general, effects of ADI were reduced when site was included as a covariate in linear models, with a larger number of significant effects observed using the Z-score deviations. Discrimination (Table S5) did not show an association with cortical thickness but was associated with amygdala volume only for the standard linear regressions with and without site as a covariate. Further, state-level racism (Table S6) was associated with thickness in several brain regions but this did not persist once adjusting for site. However, state-level racism showed a relationship with population-level normed Z-score deviations in an opposite direction. We re-ran the regression models to investigate potential associations between Z-scored deviations and individual/neighborhood factors separately within Black and white participants (Tables S7–S10). Within-group analyses revealed differential associations between the z-scored deviations in thickness and socioeconomic factors such as income, ADI, and state-level racism that survived FDR correction. Thus, socioeconomic indicators are associated with the structure of threat neurocircuitry during adolescence.

**Fig. 3:**
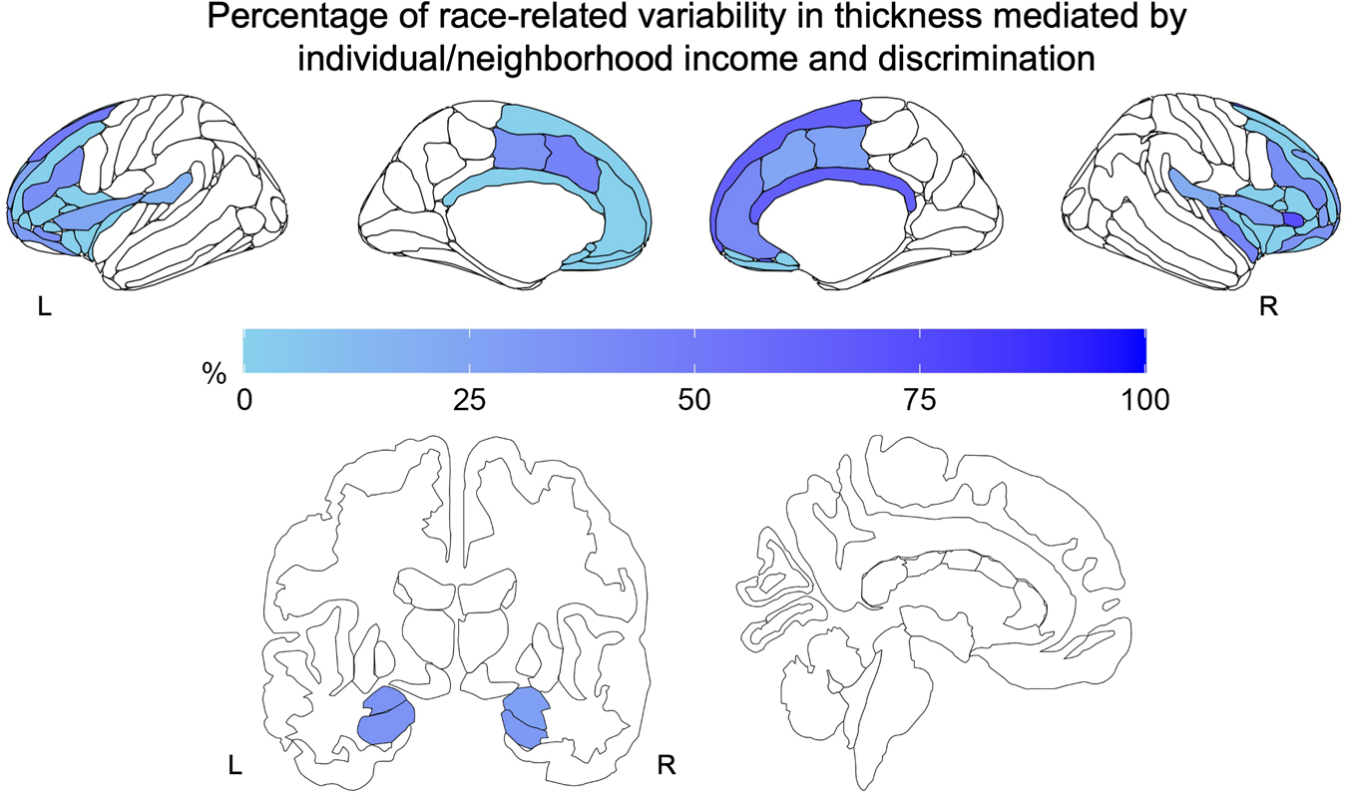
Racial inequities partially mediated race-related differences in cortical thickness of threat neurocircuitry. Parallel mediation models revealed partial or full mediation of race-related differences by socioeconomic factors in several threat-related brain regions. Colors represent percentage of the total effect accounted for by the combination of income, neighborhood disadvantage (i.e., ADI), discrimination, and state-level racism. Light colors represent lower percentage while darker colors represent a higher percentage. L = Left, R = Right.

Finally, we sought to determine if racial inequities in the individual and neighborhood factors explained race-related differences in cortical thickness (Fig. 3). Parallel mediation models that controlled for age and sex routinely demonstrated significant partial and full mediations across many brain regions (Table 2). The indirect effects of individual and neighborhood inequities accounted for between 10 and ~100% of the race-related differences in adolescent cortical thickness depending on brain region. Given significant findings using the population-level z-score deviations, we repeated the mediation analysis using the z-score deviations as well (Fig. S1, Table S8). The findings were relatively similar however we also observed patterns of inconsistent mediation and suppression effects. The present results demonstrate that significant components of race-related differences in brain structure during adolescence are driven by broad structural inequities.

**Table 2:** Parallel mediation of race-related variability by racial inequities

	Left Hemisphere							Right Hemisphere						
Region	Total Effect ©	*p*	Total Indirect Effect (ab)	*p*	Direct Effect (c’)	*p*	Percentage mediated (%)	Total Effect ©	*p*	Total Indirect Effect (ab)	p	Direct Effect (c’)	*p*	Percentage mediated (%)
G.S_frontomargin_thickness	0.115	<0.001	0.037	0.004	0.004	<0.001	32.206	0.126	<0.001	0.036	0.007	0.089	<0.001	28.846
G.S_transv_frontopol_thickness	0.025	0.095	0.02	0.14	0.005	0.821	.	0.018	0.205	0.039	0.003	–0.021	0.293	.
G.S_cingul.Ant_thickness	0.002	0.872	0.022	0.086	−0.002	0.294	.	0.07	<0.001	0.028	0.032	0.042	0.041	40.277
G.S_cingul.Mid.Ant_thickness	0.109	<0.001	0.049	<0.001	0.06	0.016	45.101	0.146	<0.001	0.035	0.005	0.111	<0.001	24.232
G.S_cingul.Mid.Post_thickness	0.113	<0.001	0.039	0.003	0.074	<0.001	34.525	0.123	<0.001	0.031	0.015	0.092	<0.001	25.441
G_front_inf.Opercular_thickness	−0.087	<0.001	0.007	0.592	−0.094	<0.001	.	−0.089	<0.001	0.026	0.052	–0.115	<0.001	.
G_front_inf.Orbital_thickness	0.083	<0.001	0.03	0.023	0.052	0.008	36.91	0.033	0.02	0.01	0.449	0.023	0.231	.
G_front_inf.Triangul_thickness	0.02	0.178	0.009	0.506	0.011	0.587	.	0.016	0.268	0.005	0.725	0.012	0.549	.
G_front_middle_thickness	0.017	0.242	0.029	0.029	−0.013	0.523	.	0.022	0.123	0.023	0.088	–0.001	0.944	.
G_front_sup_thickness	0.02	0.169	0.047	0.001	−0.027	0.182	.	0.055	<0.001	0.035	0.008	0.02	0.312	64.055
G_Ins_lg.S_cent_ins_thickness	−0.004	0.792	−0.014	0.281	0.01	0.54	.	−0.028	0.039	0.013	0.317	–0.041	0.026	.
G_insular_short_thickness	0.033	0.019	−0.001	0.926	0.034	0.065	.	−0.029	0.027	0.005	0.689	–0.034	0.062	.
G_orbital_thickness	0.093	<0.001	0.027	0.046	0.066	0.001	28.671	0.096	<0.001	0.033	0.012	0.063	0.002	34.573
G_rectus_thickness	0.067	<0.001	−0.01	0.423	0.077	<0.001	.	0.037	0.011	0.02	0.131	0.017	0.375	.
G_subcallosal_thickness	−0.007	0.603	−0.015	0.233	0.008	0.654	.	0.028	0.038	−0.006	0.667	0.034	0.074	.
S_circular_insula_ant_thickness	0.177	<0.001	0.024	0.064	0.153	<0.001	.	0.173	<0.001	0.012	0.362	0.161	<0.001	.
S_circular_insula_inf_thickness	0.032	0.026	0.033	0.009	−0.001	0.957	103.194	0.064	<0.001	0.028	0.032	0.036	0.059	43.139
S_circular_insula_sup_thickness	0.178	<0.001	0.042	0.001	0.136	<0.001	23.445	0.152	<0.001	0.043	0.001	0.109	<0.001	28.47
S_front_inf_thickness	0.102	<0.001	0.038	0.004	0.065	0.001	36.809	0.094	<0.001	0.034	0.009	0.059	0.002	36.604
S_front_middle_thickness	0.105	<0.001	0.026	0.042	0.079	<0.001	24.496	0.142	<0.001	0.039	0.002	0.103	<0.001	27.575
S_front_sup_thickness	0.094	<0.001	0.046	<0.001	0.048	0.015	48.729	0.0985	<0.001	0.021	0.108	0.074	<0.001	.
S_orbital_lateral_thickness	0.077	<0.001	0	0.985	0.077	<0.001	.	0.1	<0.001	0.026	0.043	0.074	<0.001	26.071
S_orbital_med.olfact_thickness	0.009	0.507	−0.007	0.593	0.016	0.402	.	−0.02	0.169	0.005	0.687	–0.025	0.2	.
S_orbital.H_Shaped_thickness	0.034	0.022	0.037	0.004	−0.003	0.866	109.709	0.095	<0.001	0.019	0.151	0.076	<0.001	.
S_suborbital_thickness	−0.028	0.056	−0.005	0.691	−0.023	0.244	.	0.013	0.357	0.006	0.634	0.007	0.71	.
Lat_Fis.ant.Horizont_thickness	0.062	<0.001	0.021	0.094	0.041	0.031	.	0.039	0.01	0.029	0.029	0.01	0.609	73.527
Lat_Fis.ant.Vertical_thickness	−0.042	0.005	0.022	0.096	−0.064	0.002	.	−0.028	0.058	0.023	0.073	–0.051	0.008	.
Lat_Fis.post_thickness	0.179	<0.001	0.028	0.031	0.151	<0.001	15.468	0.183	<0.001	0.038	0.002	0.145	<0.001	20.622
S_pericallosal_thickness	−0.015	0.295	−0.001	0.942	−0.014	0.486	.	−0.048	<0.001	−0.031	0.019	−0.018	0.352	63.597
Amygdala	0.196	<0.001	0.068	<0.001	0.129	<0.001	34.452	0.189	<0.001	0.052	<0.001	0.137	<0.001	27.483
Hippocampus	0.22	<0.001	0.076	<0.001	0.144	<0.001	34.464	0.23	<0.001	0.072	<0.001	0.158	<0.001	31.19

Brain region labels are short name (G = Gyrus, S = Sulcus, Lat = Lateral, Med = Medial). Percent mediated is presented only for regions which showed partial or full mediation.

## Discussion

Characterization of how racial inequities differentially impact the developing brain is crucial for a complete understanding of typical neurodevelopment and brain-based psychiatric disorders. Here, we investigated associations between race-related stressors and cortical thickness in threat neurocircuitry that support emotional processes and trauma-related disorders. Race-related differences were observed in several areas of threat neurocircuitry including the dorsal ACC, dorsolateral PFC, hippocampus, and amygdala. Individual and neighborhood level race-related stressors were also associated with cortical thickness, and inequities in these factors partially mediated race-related differences in cortical thickness. Importantly, when using normative models, we continued to observe race-related and socioeconomic-related variability in cortical thickness with some effects being larger and more diffuse across threat neurocircuitry. Together, the present study highlights that racial inequities contribute to race-related variability in cortical development and the choice of reference population impacts whether or not such effects are observable.

Income and neighborhood disadvantage (i.e., ADI) were associated with cortical thickness with and without adjusting for recruitment site in the present analyses. Prior work in the ABCD study and other datasets suggest greater family income is related to greater gray matter volume within threat neurocircuitry^13,21^ and is a known protective factor against adult psychiatric disease^29^. Further, prior work in trauma-exposed and typical adults suggests neighborhood disadvantage is associated with alterations in the functional activity and connectivity of the amygdala and dorsal ACC^16,30–32^. We also observed that youth discrimination was significantly associated with amygdala volume. Prior findings regarding an association between racial discrimination and structure of the amygdala are mixed. Amygdala volume was not associated with racial discrimination in adult trauma-exposed Black women^18^ however discrimination was associated with resting-state amygdala connectivity and signal amplitude in separate studies^17,33^. The amygdala is thought to a play an important role in threat learning and expression of the emotional response with the dorsal ACC able to exacerbate the amygdala-mediated response by supporting attentional vigilance towards potentially biologically salient stimuli in the environment^34,35^. The dorsal ACC is also highly connected with the insula and together are thought to comprise the brain’s “salience network”^36,37^, and connectivity between the amygdala and the salience network is modulated by combinatorial effects of trauma and neighborhood disadvantage^30,31^. As neighborhood disadvantage partially represents unsafe and stressful environments, it may be that greater neighborhood disadvantage during development contributes to a heightened vigilance to potential threats in the environment via modulation of amygdala-salience network structure and function. The enhanced threat detection may ultimately be beneficial and serve to regulate (i.e., diminish) the emotional response to threat by increasing anticipatory processes (e.g., conditioned diminution)^7,16^. Future work is needed to determine if the functional architecture of Black and white children in the ABCD and other studies show neighborhood disadvantage-related shifts and if these are exacerbated by chronic exposure to such adversities.

It is somewhat surprising, given the present findings and prior literature on discrimination, that state-level racism in the present sample was more robustly associated with cortical thickness compared to discrimination. Prior work observed that state-level racism was negatively associated with hippocampal volume^4^. In the present work, state-level racism was only positively associated with hippocampal volume in the Z-Score deviation models however we note there was a nonsignificant negative correlation in the standard general linear models (Table S6). It is tempting to speculate that the results could suggest, at the current developmental stage, that neighborhood metrics of discrimination – potentially indicative of general societal and institutional practices – have unique effects on cortical morphology compared to individual racial discrimination. Prior work has previously shown neighborhood level metrics of poverty have unique associations with morphology compared to individual metrics such as income-to-needs ratios^15^. A speculative hypothesis would be that structural/institutional racism may have potentially more impact on “top-down” regulatory circuitry while individual discrimination has more “bottom-up” effects. While such a hypothesis is partially in line with previous research on structural versus individual racism (see^38^ for review), we also caution that the state-level racism measure employed in the present models is highly collinear with geospatial factors like neuroimaging site (R^2^ = 0.99). Associations between morphology with state-level racism and brain morphology were largely attenuated in linear models that covaried for site, and largely reversed in the Brainchart Deviation models (which implicitly adjusts for site). It is therefore difficult to fully discern the mechanisms underlying the *unique* associations with state-level racism in the present study and further research is needed to understand this association.

Parallel mediation models revealed that variability in cortical thickness within threat neurocircuitry between Black and white adolescents was accounted for by inequities in individual and neighborhood level factors. Further, we observed several complete mediations of race-related differences (significant total and indirect, but not direct, paths) in subregions of the dorsolateral and ventrolateral PFC as well as the insula. Although the percentage of race-related effects was not 100% for each region, the appearance of residual race-related variance is unsurprising given the selection of factors used in the present analysis. The income and neighborhood level factors are fairly coarse snapshots of individuals’ lived experience that may not fully capture important nuances such as the severity or chronicity of other events tied to these factors. For example, lower income may prevent a child from participating in extracurricular activities that then impact other aspects of their life. Recent evidence further suggests that race-related stressors can have effects prenatally and resultant effects on infant development may be non-linear^39^. In the present study, parts of the insular and orbital sulci showed percentages of the total effect mediated by individual and neighborhood factors over 100% (i.e., *ab* path greater than the total effect) suggesting there are likely interactions (i.e., non-linear associations) among the variables related to race-related differences in neurobiology. Similar results were obtained when using the z-scored deviations from the population-level normative models, though we also observed more instances of inconsistent mediation and suppression effects again in insular and orbital regions (e.g., negative percent mediated) (Table S11). Suppression effects occur when the presence of the mediator serves to enhance the effect of the predictor variable on the outcome variable^40^. The findings may suggest that, within these regions, race-related variability is larger once accounting for inequities and such an effect may again be driven by unaccounted for third variables, though the specific mechanism is not clear. Therefore, further consideration of how race-related stressors interact over time may lead to increased understanding of how race-related variability in brain development manifests.

The present findings highlight that modeling choice plays a significant role in whether associations with race or socioeconomic-related variability with neural structure are observable. Inclusion of site as a factor in general linear models led to fewer associations between cortical thickness and race, income, ADI, and state-level racism. Participants across each recruitment site differed in each of the assessed individual and neighborhood level factors. Further, state-level racism was highly collinear with the site variable (R^2^ = 0.99) suggesting similar information may be captured for both measures and lowering the unique variance of either variable in the models. The multicollinearity highlights an important problem with site-wise adjustments in neuroimaging investigations in that the presumed “noise” of between site variance may capture important sociodemographic factors of interest. Residential segregation exists among racial groups throughout the U.S. and is tied to inequitable access to resources^1,41^. The sociohistorical reasons for persistent racial segregation and socioeconomic disparities within the U.S. are not random and have major influence on the lives of minoritized individuals^42^ which, in turn, may influence neurobiology^43^. Careful consideration is thus needed when investigating sociodemographic associations with brain biology as adjusting for geographic location may unintentionally and artificially reduce the effects of racial inequities. New approaches for removing site effects from neuroimaging data while retaining important sources of individual variation are currently in development that may provide solutions for the current problem^44^. Nevertheless, care and consideration is necessary for the generation of ecologically valid models of neurobiology.

Although accounting for site within linear models reduced the appearance of race-related differences in cortical thickness, race- and socioeconomic-related differences were largely maintained when using deviations derived from the normative models. The findings suggest that the normative modeling approach may be effective at accounting for site variance while preserving collinear effects related to important sociodemographic factors. The findings from the population-level normative model may also indicate that differences between white and Black children in cortical thickness are not very large relative to the global population. Indeed, standardized estimates from the mediation models indicate the total magnitude of the race-related effects to be relatively minor ( < 1/5 of a standard deviation). Such an interpretation is consistent with the fact that race has little biological meaning and re-emphasizes that neural differences between racial groups are minor. However, subsequent models tested within racial group (i.e., only in white or in Black participants) revealed mixed – and somewhat surprising - effects in terms of associations between cortical thickness and socioeconomic factors. For example, the association between cortical thickness and state-level racism was largely driven by associations observed in white participants. As noted in the initial validation studies, normative models were generated on predominately “WEIRD” (i.e., Western, Educated, Industrialized, Rich, and Democratic) samples that may impact generalizability of the approach^26^ and may partially explain the discrepancy between within-group results. Emergent research suggests normative modeling approaches can identify deviations in brain structure in significant neuropsychiatric conditions such as schizophrenia^26,27,45^. Nevertheless, psychosis and schizophrenia also have significant race-related variability that is intimately tied to racial inequities^46^. Therefore, while normative modeling and population-based approaches are likely beneficial for some research questions, it may be pertinent to strongly consider both the potential for an influence of racial inequities on the phenotype of interest as well as which group of individuals is established as the “norm” for generation of deviations.

We further emphasize that the implications raised here about the inclusion of site and the use of population-based methods in the investigation of race-related stressors are not esoteric and have important consequences for research on emotional health in adolescence. Threat neurocircuitry is known to the play a significant role in a number of neuropsychiatric disorders including major depression^47^, anxiety^48^, and posttraumatic stress disorder^34^. Significant research over the past decade has thus suggested that evaluating such circuitry may provide neural signatures for early identification of psychiatric disorders such as PTSD^34,49–51^. However, the predictive utility of such neural signatures can differ widely by racial group as a direct result of structural inequity within the U.S^30^. Thus, failure to appropriately integrate inequity-related variance in neurobiological models of development and disease risks the generation of ungeneralizable treatment and intervention targets. It is therefore critical that neuropsychiatric research carefully investigate the usage and generalizability of models built on population-based methods to ensure important sources of variability relevant to psychiatric disease are not lost and that models can be equitably applied across all individuals.

The present study has several limitations to consider. Given the current normative models, it was not possible to use a longitudinal approach for the MRI data. The normative models employed here were derived from a large sample of ~57 K participants which included ABCD study participants’ baseline MRI data. Generating deviation scores from the baseline data for the current participants may bias model performance for the present analysis. An alternative approach, such as Gaussian Process Regression^27^, is available to generate normative models using the baseline data to then apply to the follow-up data. However, the age-range for each assessment timepoint is limited to ~2-years and does not offer sufficient variability to fully account for potential age-related associations compared to the previously defined model. We further note that, given that the data here is cross-sectional, the directionality of associations between thickness and variables of interest cannot be conclusively established. Specifically, although we interpret these findings as socioeconomic variables contribute to differences in thickness, it is possible that the inverse relationship could be true. However, the fact that all socioeconomic variables necessarily predated the imaging data—and the imaging data cannot reasonably affect the socioeconomic variables (e.g., children cannot choose state-levels of racism)—we contend the most reasonable interpretation is the one we proposed. We also note that the measures employed here likely do not fully capture racialized experiences within the U.S. For example, the discrimination measure employed in the ABCD Study is not a validated instrument, and other measures such as the Experiences of Discrimination or Everyday Discrimination Scale may be better suited to understanding perceived discrimination^52,53^. The present analysis was also focused on threat neurocircuitry that is strongly implicated in internalizing psychopathology and PTSD. Emergent evidence suggests that PTSD development may be tied to variation in connections between threat and sensorial circuitry^54,55^. Childhood stress and adversity appears to also have effects on sensory circuits, such as ventral visual stream, that undergo development before association cortices^56–59^. Research on the potential effects of racialized stress and inequity on sensorial circuitry may be a valuable avenue for future research. We also emphasize that the present analysis is limited to white and Black children for whom parents identified as a single racial identity. It may therefore be difficult to fully generalize the findings to all racially minoritized groups. Other racial or multiracial identities may have important and/or unique interactions within society that could differentially affect neurobiology in complex ways. Granular investigations on the unique effects of ethnicity and race-related stressors on the brain should receive further attention with guidance from those with significant expertise in respective areas that is not available in the present investigation.

In conclusion, race-related variability in cortical thickness of threat neurocircuitry during early adolescence is due – in part – to inequities in individual and society-level factors of economic resources and racial prejudice. Although race-related variability is apparent regardless of geographical location in the sample, model choice plays a role in the strength of associations observed between race/socioeconomic factors and cortical thickness. However, the normative models revealed associations between cortical thickness and sociodemographic factors independently that were stronger than those observed when covarying for site. Thus, population-based norming may be a useful approach for quantifying sociodemographic variability within samples that are critical to our understanding of neural development and psychopathology.

## Methods

Data were drawn from the 4.0 release of the Adolescent Brain and Cognitive Development (ABCD) study. The data collection protocol for the ABCD study has been described elsewhere and we therefore only briefly summarize key points within the methods^60^. Data were accessed from the NIMH Data Archive from 05/01/2022 through 06/15/2023. Participants from the ABCD were recruited from 21 research sites across the U.S. and primarily from public and private school systems within the 21 catchment areas. The 4.0 release of the ABCD includes two-year follow-up data from a subset of participants. The initial normative modeling approach included participants from the baseline MRI scans for ABCD participants and thus it would be inappropriate to apply these models to the baseline MRI data sample. Therefore, for the present analyses, we utilized data from the two-year follow-up for MRI analyses. A final sample of 6505 participants (*n* = 3538 female, M = 11.94, SD = 0.64 years of age) were retained following removal of participants without MRI data at the two-year follow-up or corresponding socioeconomic variables (described below). The present use of data was approved by the Mass General Brigham Institutional Review Board. All ethical regulations relevant to human research participants were followed.

### Race/ethnicity

The present manuscript was primarily focused on differences in threat neurocircuitry between Black and white participants in the US. Racial group was reported by parents at the initial visit. Parents selected from 16 options (e.g., white, Black/African-American, Alaska Native, etc.) with multiple selections allowed (NDA: pdem02). We selected participants whose parents identified their child as only Black or white. Ethnicity was not exclusionary in the present analysis to maintain representation of Hispanic/Latinx white and Black children. Normative models (described below) were applied to all participants and outcome measures were retained in analyses only for participants of interest.

### Individual and neighborhood-level socioeconomic variables

Measurements of participant income and neighborhood disadvantage are described in prior work. Briefly, parents self-reported incomes for themselves and/or their partner across 10 levels ( < $5000 to ≥$200,000). The combined income of the caregiver and partner was used as the index of income (NDA: abcdlpds01 & pdem02). Neighborhood disadvantage was operationalized using the Area Deprivation Index (ADI). Briefly, data from the American Community Survey comprising measures of income, education, employment, and housing quality from U.S. census tracts were combined to derive a weighted sum score then normalized to a scale of 0–100 ^61,62^ (NDA: abcd_rhds01).

Experiences of discrimination were assessed with the 11-item youth discrimination measure created for the ABCD study (NDA: abcd_ydmes01). Youth self-reported whether they had experienced discrimination related to race/ethnicity, immigrant status, sexual orientation, and/or weight in the past twelve months (dim_yesno_q1 through q4). Youth also indicated how often they felt treated unfairly by teachers, other adults outside school, or other students, that they were treated unfairly due to their ethnic group (dim_matrix_q1 through dim_matrix_q4). They also indicated how often they felt unwanted in American society, felt unaccepted by other Americans, or felt that other Americans were against them (dim_matrix_q5 through dim_matrix_q7). Past twelve month discrimination was endorsed dichotomously as yes or no, with feelings related to discrimination assessed on a 5 point Likert scale from almost never to very often. For the present analyses, we focused on racial discrimination and summed scores across the seven questions on subjective feelings to derive a perceived discrimination score (i.e., dim_matrix_q1 through dim_matrix_q7). State level indicators of racism were included as part of the 4.0 release and constructed as previously described^4^. Briefly, 31 items related attitudes towards race were taken from three separate sources (Project Implicit, the General Social Survey, and the American National Election Survey) and entered into a factor analysis to derive individual measures of racial prejudice towards Black individuals (NDA: abcd_rhds01).

For the present analyses, individual socioeconomic factors (i.e., income and discrimination) at the two-year follow-up were used while neighborhood socioeconomic factors (i.e., ADI and state-level racism) at baseline were used. Discrimination was not initially assessed until the one-year follow-up and thus there was no baseline data available. Further, only baseline state-level indicators of racism are available in the 4.0 release of the dataset. Therefore, to keep factors largely temporally congruent, we used the two-year follow-up assessments for the individual factors and the initial baseline assessments for neighborhood factors.

### Magnetic resonance imaging

Structural MRI data were collected across the 21 sites on Siemens Prisma, General Electric 750, and Philips 3-T scanners, using prospective motion correction when available. Detailed information on imaging protocols, parameters, and processing of the structural imaging data has been published elsewhere^63,64^. Briefly, structural MRI (T1-weighted and T2-weighted) data were preprocessed by the ABCD team using FreeSurfer, version 5.3.0 (https://surfer.nmr.mgh.harvard.edu). Images were corrected for gradient nonlinearity distortions, intensity inhomogeneities, and head motion and resampled into alignment with an averaged reference brain. The cortical surface was then reconstructed, and subcortical regions of the brain were segmented. To harmonize with prior normative modeling approaches^24–26^, cortical thickness of a priori regions of threat neurocircuitry were selected from the Destrieux parcellation and gray matter volume for the amygdala and hippocampus were extracted from anatomic segmentation (NDA: abcd_mrisdo10201 & abcd_asmrip10201). Specific regions (n = 31) selected for analysis that covered the PFC, amygdala, hippocampus, and insula for the left and right hemisphere separately are listed in the supplement (Table S1). Participants whose MRI data failed T1 quality-control checks (NDA: mriqcrp10301) were excluded from the analyses. In total, gray matter volumes of 114 regions of interest were included in the statistical analyses.

### Statistics and reproducibility

Analyses were completed using a combination of R and JASP. Demographic characteristics (i.e., age, sex, income, ADI, discrimination, state-level racism) were compared between white and Black participants with Chi-Square and t-tests with adjustments for inequality of variance as appropriate. We also completed general linear models to assess differences in income, ADI, discrimination, and state-level racism between the recruitment sites. We first performed initial mass univariate analyses to investigate race-related differences in cortical thickness and subcortical volume including covariates for age and sex. Each brain parcel (n = 31) was tested separately for the left and right hemisphere (62 total models). A sensitivity analysis was further conducted accounting for ABCD scan site (NDA: abcd_lt01). The models were then repeated without race and instead including income, discrimination, ADI, and state-level racism as independent variables within a single model. False discovery rate (FDR) correction for the number of brain regions tested was done using the Benjamini-Hochberg approach. FDR correction was applied separately for each predictor of interest (see Supplementary Note). Effects were considered significant at q < 0.05.

We then applied pretrained population-level normative models that quantify the typical ranges of cortical thickness and subcortical volumes across the lifespan described previously^24,25^ (Google Collab link provided below) and freely available using the PCNToolkit (https://github.com/predictive-clinical-neuroscience/braincharts) to derive participant-level standardized deviations of brain morphology from the population normative values. The resultant Brainchart Deviation scores were then used in linear regression analyses to investigate either race-related effects or effects of income, discrimination, ADI, and state-level racism (identical to models above excluding covariates for age and sex). Age, sex, and scan site were not included in analyses of the Brainchart Deviations, as the current normative model approach used adjusts for these covariates in computing the deviations. Again, false discovery rate correction using the Benjamini-Hochberg approach was employed separately for each predictor of interest across brain regions and considered significant at q < 0.05.

Finally, we completed parallel mediation analyses (*lavaan*) to determine if individual and neighborhood factors significantly mediated race-related differences in threat neurocircuitry while covarying for age and sex. Outcome variables for these analyses were the original cortical thickness and subcortical volume data for each participant. We further calculated the proportion of race-related variance mediated by the individual and neighborhood factors by multiplying the dividend of the indirect effect over the total effect by a hundred (i.e., ab/c * 100).

### Reporting summary

Further information on research design is available in the Nature Portfolio Reporting Summary linked to this article.

### Supplementary information

Supplementary information

Reporting Summary

## Data Availability

The ABCD Study 4.0 release is a publicly available dataset accessible with a data use agreement from the NIMH Data Archive. The Predictive Neuroscience Toolkit and associated normative models are freely available via GitHub (https://github.com/predictive-clinical-neuroscience) and Google Collab (https://colab.research.google.com/github/predictive-clinical-neuroscience/braincharts/blob/master/scripts/apply_normative_models_ct.ipynb). Template JupyterNotebook and R Studio code for the present analyses is available via GitHub (https://github.com/nateharnett/ABCD_BrainDeviation_Analysis) and upon request. Data used in the preparation of this article were obtained from the Adolescent Brain Cognitive Development (ABCD) Study (https://abcdstudy.org), held in the NIMH Data Archive. This is a multisite longitudinal study designed to recruit more than 10,000 children ages 9–10 and follow them over 10 years into early adulthood. The ABCD Study is supported by NIH and additional federal partners under award numbers U01DA041022, U01DA041028, U01DA041048, U01DA041089, U01DA041106, U01DA041117, U01DA041120, U01DA041134, U01DA041148, U01DA041156, U01DA041174, U24DA041123, and U24DA041147. A full list of supporters is available at https://abcdstudy.org/nih-collaborators. A listing of participating sites and a complete listing of the study investigators can be found at https://abcdstudy.org/principal-investigators.html. ABCD consortium investigators designed and implemented the study and/or provided data but did not necessarily participate in the analysis or writing of this report. This report reflects the views of the authors and may not reflect the opinions or views of the NIH or ABCD consortium investigators. The ABCD data repository grows and changes over time. The ABCD data used in this report came from 10.15154/1523041.
