# Supplementary material for: Population-level normative models reveal race- and socioeconomic-related variability in cortical thickness of threat neurocircuitry
Source: Commun Biol. 2024 Jun 19;7:745. doi: 10.1038/s42003-024-06436-7 (PMC11187116; doi:10.1038/s42003-024-06436-7)
Supplement: Supplementary file 1 — Supplementary information [file 42003_2024_6436_MOESM1_ESM.pdf]

Supplemental Material to:

Population-level normative models attenuate race- and socioeconomic-related variability in cortical thickness of threat neurocircuitry.

Nathaniel Harnett[1,2,*], Negar Fani[3], Grace Rowland[1], Poornima Kumar[1,2,4], Saige Rutherford[5,6,7],

& Lisa D. Nickerson[2,4,8]

[1]Division of Depression and Anxiety, McLean Hospital, Belmont, USA
[2]Department of Psychiatry, Harvard Medical School, Boston, USA
[3]Department of Psychiatry and Behavioral Neuroscience, Emory University, Atlanta, USA
[4]McLean Imaging Center, McLean Hospital, Belmont, USA
[5]Department of Cognitive Neuroscience, Radboud University Nijmegen Medical Centre, Nijmegen, Netherlands
[6]Donders Institute, Radboud University Nijmegen, Nijmegen, Netherlands
[7]Department of Psychiatry, University of Michigan-Ann Arbor, Ann Arbor, United States
[8]Applied Neuroimaging Statistics Research Laboratory, McLean Hospital, Belmont, USA

Table S1.

**Hemi**	**Label**	**Division**
lh/rh	_G_front_sup_thickness	Dorsolateral PFC
lh/rh	_S_front_inf_thickness	Dorsolateral PFC
lh/rh	_S_front_middle_thickness	Dorsolateral PFC
lh/rh	_G_front_inf.Opercular_thickness	Dorsolateral PFC
lh/rh	_G_front_middle_thickness	Dorsolateral PFC
lh/rh	_G.S_cingul.Mid.Ant_thickness	Dorsomedial PFC
lh/rh	_S_orbital_med.olfact_thickness	Dorsomedial PFC
lh/rh	_G.S_cingul.Ant_thickness	Dorsomedial PFC
lh/rh	_G.S_cingul.Mid.Post_thickness	Dorsomedial PFC
lh/rh	_S_front_sup_thickness	Dorsomedial PFC
lh/rh	_S_circular_insula_ant_thickness	Insula
lh/rh	_G_Ins_lg.S_cent_ins_thickness	Insula
lh/rh	_S_circular_insula_inf_thickness	Insula
lh/rh	_G_insular_short_thickness	Insula
lh/rh	_S_circular_insula_sup_thickness	Insula
lh/rh	_G.S_frontomargin_thickness	Ventrolateral PFC
lh/rh	_G_front_inf.Orbital_thickness	Ventrolateral PFC
lh/rh	_G_front_inf.Triangul_thickness	Ventrolateral PFC
lh/rh	_S_orbital.H_Shaped_thickness	Ventrolateral PFC
lh/rh	_Lat_Fis.ant.Horizont_thickness	Ventrolateral PFC
lh/rh	_Lat_Fis.ant.Vertical_thickness	Ventrolateral PFC
lh/rh	_Lat_Fis.post_thickness	Ventrolateral PFC
lh/rh	_S_orbital_lateral_thickness	Ventrolateral PFC
lh/rh	_G.S_transv_frontopol_thickness	Ventromedial PFC
lh/rh	_G_orbital_thickness	Ventromedial PFC
lh/rh	_G_rectus_thickness	Ventromedial PFC
lh/rh	_G_subcallosal_thickness	Ventromedial PFC
lh/rh	_S_suborbital_thickness	Ventromedial PFC
lh/rh	_S_pericallosal_thickness	Ventromedial PFC
Left/Right	Amygdala	Subcortical
Left/Right	Hippocampus	Subcortical

Table S2. Race-related variability in cortical thickness.

Region	Left Hemisphere *GLM (Age + Sex)*		*GLM (Age + Sex + Site*		*Brainchart Deviatoins*		Right Hemisphere *GLM (Age + Sex)*		*GLM (Age + Sex + Site*		*Brainchart Deviatoins*	
	Estimate (β)	FDR (p)	Estimate (β)	FDR (p)	Estimate (β)	FDR (p)	Estimate (β)	FDR (p)	Estimate (β)	FDR (p)	Estimate (β)	FDR (p)
G.S_frontomargin_thickness	0.108	0.000	0.092	0.000	0.099	0.000	0.136	0.000	0.101	0.000	0.103	0.000
G.S_transv_frontopol_thickness	0.017	0.194	0.011	0.486	0.014	0.318	0.011	0.401	-0.008	0.578	-0.012	0.389
G.S_cingul.Ant_thickness	0.001	0.948	-0.011	0.435	0.000	0.990	0.081	0.000	0.017	0.237	0.027	0.047
G.S_cingul.Mid.Ant_thickness	0.113	0.000	0.095	0.000	0.112	0.000	0.163	0.000	0.123	0.000	0.120	0.000
G.S_cingul.Mid.Post_thickness	0.114	0.000	0.110	0.000	0.110	0.000	0.144	0.000	0.122	0.000	0.115	0.000
G_front_inf.Opercular_thickness	-0.108	0.000	-0.102	0.000	-0.100	0.000	-0.100	0.000	-0.092	0.000	-0.083	0.000
G_front_inf.Orbital_thickness	0.090	0.000	0.086	0.000	0.077	0.000	0.032	0.014	0.023	0.126	0.024	0.071
G_front_inf.Triangul_thickness	0.013	0.325	0.000	0.993	0.005	0.781	-0.001	0.948	-0.004	0.813	0.001	0.978
G_front_middle_thickness	0.020	0.137	-0.009	0.535	0.010	0.463	0.017	0.211	-0.016	0.299	-0.002	0.884
G_front_sup_thickness	0.009	0.499	-0.030	0.029	-0.020	0.139	0.051	0.000	-0.004	0.813	0.003	0.856
G_Ins_lg.S_cent_ins_thickness	0.003	0.842	-0.014	0.332	0.003	0.856	-0.027	0.038	-0.043	0.002	-0.024	0.073
G_insular_short_thickness	0.033	0.010	0.017	0.247	0.024	0.073	-0.037	0.004	-0.045	0.001	-0.018	0.176
G_orbital_thickness	0.099	0.000	0.097	0.000	0.100	0.000	0.100	0.000	0.091	0.000	0.091	0.000
G_rectus_thickness	0.069	0.000	0.036	0.008	0.026	0.056	0.040	0.002	0.028	0.045	0.024	0.075
G_subcallosal_thickness	0.001	0.948	-0.026	0.053	0.034	0.011	0.040	0.002	0.010	0.486	0.049	0.000
S_circular_insula_ant_thickness	0.204	0.000	0.183	0.000	0.172	0.000	0.182	0.000	0.164	0.000	0.151	0.000
S_circular_insula_inf_thickness	0.045	0.000	0.050	0.000	0.059	0.000	0.074	0.000	0.074	0.000	0.072	0.000
S_circular_insula_sup_thickness	0.200	0.000	0.176	0.000	0.171	0.000	0.163	0.000	0.152	0.000	0.134	0.000
S_front_inf_thickness	0.123	0.000	0.092	0.000	0.126	0.000	0.098	0.000	0.053	0.000	0.081	0.000
S_front_middle_thickness	0.116	0.000	0.071	0.000	0.105	0.000	0.157	0.000	0.066	0.000	0.099	0.000
S_front_sup_thickness	0.105	0.000	0.048	0.000	0.085	0.000	0.114	0.000	0.029	0.030	0.065	0.000
S_orbital_lateral_thickness	0.084	0.000	0.070	0.000	0.091	0.000	0.120	0.000	0.086	0.000	0.108	0.000
S_orbital_med.olfact_thickness	0.017	0.207	0.001	0.931	0.016	0.226	-0.002	0.932	-0.044	0.001	-0.039	0.003
S_orbital.H_Shaped_thickness	0.025	0.052	0.003	0.822	0.017	0.213	0.105	0.000	0.051	0.000	0.057	0.000
S_suborbital_thickness	-0.040	0.002	-0.063	0.000	-0.057	0.000	0.014	0.296	-0.022	0.130	-0.032	0.016
Lat_Fis.ant.Horizont_thickness	0.062	0.000	0.045	0.001	0.058	0.000	0.038	0.003	0.015	0.309	0.032	0.015
Lat_Fis.ant.Vertical_thickness	-0.054	0.000	-0.063	0.000	-0.038	0.004	-0.029	0.027	-0.035	0.013	-0.018	0.190
Lat_Fis.post_thickness	0.197	0.000	0.206	0.000	0.180	0.000	0.200	0.000	0.202	0.000	0.183	0.000
S_pericallosal_thickness	-0.023	0.087	-0.007	0.669	0.002	0.884	-0.057	0.000	-0.047	0.001	-0.030	0.026
Amygdala	0.201	0.000	0.217	0.000	0.183	0.000	0.200	0.000	0.209	0.000	0.166	0.000
Hippocampus	0.236	0.000	0.242	0.000	0.233	0.000	0.255	0.000	0.254	0.000	0.229	0.000

Table S3. Associations between income and cortical thickness.

Region	Left Hemisphere GLM (Age + Sex)		GLM (Age + Sex + Site)		Brainchart Deviatoins		Right Hemisphere GLM (Age + Sex)		GLM (Age + Sex + Site)		Brainchart Deviatoins	
	Estimate (β)	FDR (p)	Estimate (β)	FDR (p)	Estimate (β)	FDR (p)	Estimate (β)	FDR (p)	Estimate (β)	FDR (p)	Estimate (β)	FDR (p)
G.S_frontomargin_thickness	0.087	0.000	0.064	0.001	0.061	0.001	0.049	0.004	0.016	0.467	0.026	0.198
G.S_transv_frontopol_thickness	0.036	0.042	0.019	0.397	0.021	0.356	0.017	0.383	0.007	0.780	-0.002	0.951
G.S_cingul.Ant_thickness	0.004	0.879	-0.009	0.694	-0.019	0.392	0.037	0.037	0.019	0.389	0.014	0.532
G.S_cingul.Mid.Ant_thickness	0.053	0.003	0.047	0.009	0.038	0.043	0.072	0.000	0.062	0.001	0.062	0.000
G.S_cingul.Mid.Post_thickness	0.058	0.001	0.061	0.001	0.058	0.001	0.069	0.000	0.069	0.000	0.067	0.000
G_front_inf.Opercular_thickness	-0.001	0.972	0.013	0.571	0.001	0.979	0.003	0.881	0.016	0.467	0.006	0.815
G_front_inf.Orbital_thickness	0.051	0.003	0.046	0.017	0.047	0.010	-0.005	0.831	0.000	0.992	-0.011	0.629
G_front_inf.Triangul_thickness	-0.006	0.773	0.006	0.788	-0.002	0.951	0.002	0.927	0.011	0.644	-0.004	0.901
G_front_middle_thickness	-0.009	0.711	0.006	0.788	-0.020	0.367	-0.009	0.689	0.011	0.635	-0.016	0.474
G_front_sup_thickness	0.016	0.437	0.023	0.276	0.005	0.845	0.012	0.571	0.023	0.284	0.012	0.587
G_Ins_lg.S_cent_ins_thickness	0.037	0.038	0.008	0.729	0.017	0.434	0.046	0.010	0.019	0.397	0.020	0.363
G_insular_short_thickness	0.065	0.000	0.041	0.032	0.051	0.004	0.043	0.017	0.024	0.276	0.026	0.198
G_orbital_thickness	0.087	0.000	0.065	0.001	0.069	0.000	0.067	0.000	0.049	0.010	0.044	0.015
G_rectus_thickness	0.039	0.030	0.026	0.201	0.012	0.587	0.001	0.967	-0.004	0.858	-0.007	0.784
G_subcallosal_thickness	0.005	0.814	-0.009	0.694	-0.002	0.953	0.039	0.030	0.027	0.200	0.015	0.495
S_circular_insula_ant_thickness	0.078	0.000	0.052	0.006	0.063	0.000	0.068	0.000	0.046	0.017	0.052	0.004
S_circular_insula_inf_thickness	0.106	0.000	0.077	0.000	0.069	0.000	0.094	0.000	0.067	0.000	0.059	0.001
S_circular_insula_sup_thickness	0.114	0.000	0.105	0.000	0.107	0.000	0.070	0.000	0.074	0.000	0.082	0.000
S_front_inf_thickness	0.065	0.000	0.055	0.002	0.048	0.008	0.035	0.051	0.030	0.149	0.014	0.532
S_front_middle_thickness	0.029	0.115	0.037	0.054	0.014	0.532	0.031	0.080	0.016	0.467	0.012	0.587
S_front_sup_thickness	0.060	0.001	0.057	0.002	0.045	0.014	0.021	0.257	0.014	0.538	0.003	0.951
S_orbital_lateral_thickness	0.029	0.113	0.002	0.933	0.018	0.411	0.047	0.008	0.025	0.261	0.032	0.087
S_orbital_med.olfact_thickness	-0.007	0.773	0.000	0.992	0.000	0.988	-0.012	0.577	-0.011	0.635	-0.017	0.454
S_orbital.H_Shaped_thickness	0.035	0.051	0.009	0.707	0.021	0.340	0.052	0.003	0.024	0.239	0.036	0.052
S_suborbital_thickness	-0.022	0.257	-0.028	0.200	-0.040	0.029	0.007	0.773	0.004	0.856	0.001	0.979
Lat_Fis.ant.Horizont_thickness	0.051	0.003	0.045	0.020	0.050	0.006	0.023	0.222	0.021	0.353	0.024	0.250
Lat_Fis.ant.Vertical_thickness	0.012	0.577	0.003	0.920	0.007	0.812	-0.024	0.213	-0.022	0.335	-0.033	0.087
Lat_Fis.post_thickness	0.116	0.000	0.097	0.000	0.099	0.000	0.101	0.000	0.094	0.000	0.083	0.000
S_pericallosal_thickness	-0.007	0.773	-0.018	0.428	-0.035	0.062	-0.025	0.190	-0.037	0.063	-0.043	0.021
Amygdala	0.118	0.000	0.113	0.000	0.135	0.000	0.100	0.000	0.092	0.000	0.111	0.000

Hippocampus	0.158	0.000	0.154	0.000	0.145	0.000	0.142	0.000	0.138	0.000	0.132	0.000

Table S4. Associations between ADI and cortical thickness

Region	Left Hemisphere: GLM (Age + Sex)		Left Hemisphere: GLM (Age + Sex + Site		Left Hemisphere: Brainchart Deviatoins		Right Hemisphere: GLM (Age + Sex)		Right Hemisphere: GLM (Age + Sex + Site		Right Hemisphere: Brainchart Deviatoins	
	Estimate (β)	FDR (p)	Estimate (β)	FDR (p)	Estimate (β)	FDR (p)	Estimate (β)	FDR (p)	Estimate (β)	FDR (p)	Estimate (β)	FDR (p)
G.S_frontomargin_thickness	-0.029	0.122	-0.043	0.074	-0.041	0.030	-0.050	0.008	-0.087	0.000	-0.075	0.000
G.S_transv_frontopol_thickness	0.025	0.192	0.004	0.867	-0.002	0.896	-0.010	0.598	-0.039	0.104	-0.048	0.010
G.S_cingul.Ant_thickness	-0.027	0.171	-0.018	0.498	-0.056	0.003	-0.036	0.063	-0.019	0.478	-0.068	0.000
G.S_cingul.Mid.Ant_thickness	-0.069	0.000	-0.036	0.111	-0.084	0.000	-0.077	0.000	-0.054	0.021	-0.078	0.000
G.S_cingul.Mid.Post_thickness	-0.056	0.003	-0.036	0.119	-0.047	0.011	-0.067	0.000	-0.039	0.104	-0.056	0.003
G_front_inf.Opercular_thickness	0.026	0.171	0.041	0.099	0.012	0.566	0.013	0.527	0.010	0.726	-0.003	0.871
G_front_inf.Orbital_thickness	-0.026	0.171	-0.053	0.031	-0.029	0.127	-0.030	0.117	-0.038	0.119	-0.042	0.025
G_front_inf.Triangul_thickness	-0.010	0.598	0.019	0.498	0.006	0.799	0.015	0.470	0.010	0.726	-0.014	0.510
G_front_middle_thickness	-0.053	0.005	-0.008	0.771	-0.039	0.034	-0.035	0.067	0.004	0.862	-0.040	0.034
G_front_sup_thickness	-0.051	0.007	0.007	0.804	-0.046	0.013	-0.060	0.001	-0.017	0.519	-0.052	0.006
G_Ins_lg.S_cent_ins_thickness	0.045	0.018	0.014	0.576	0.008	0.731	0.041	0.031	0.047	0.057	0.012	0.583
G_insular_short_thickness	0.043	0.026	0.036	0.120	0.018	0.363	0.043	0.026	0.048	0.057	0.005	0.799
G_orbital_thickness	-0.006	0.778	-0.037	0.119	-0.029	0.122	-0.036	0.063	-0.063	0.008	-0.070	0.000
G_rectus_thickness	-0.014	0.497	-0.007	0.776	-0.033	0.080	-0.056	0.003	-0.045	0.068	-0.066	0.001
G_subcallosal_thickness	-0.003	0.846	0.024	0.309	-0.040	0.032	-0.006	0.782	0.011	0.697	-0.056	0.003
S_circular_insula_ant_thickness	-0.061	0.001	-0.092	0.000	-0.058	0.003	-0.046	0.014	-0.075	0.001	-0.058	0.003
S_circular_insula_inf_thickness	0.032	0.103	-0.010	0.726	-0.025	0.198	0.036	0.063	-0.003	0.867	-0.012	0.566
S_circular_insula_sup_thickness	-0.052	0.005	-0.043	0.070	-0.049	0.008	-0.054	0.003	-0.057	0.011	-0.052	0.005
S_front_inf_thickness	-0.073	0.000	-0.044	0.066	-0.043	0.019	-0.056	0.003	-0.045	0.064	-0.054	0.004
S_front_middle_thickness	-0.075	0.000	-0.004	0.862	-0.050	0.008	-0.075	0.000	-0.065	0.003	-0.066	0.000
S_front_sup_thickness	-0.098	0.000	-0.023	0.356	-0.038	0.043	-0.077	0.000	-0.037	0.104	-0.059	0.002
S_orbital_lateral_thickness	-0.019	0.342	-0.036	0.122	0.005	0.799	-0.030	0.121	-0.061	0.010	-0.030	0.119
S_orbital_med.olfact_thickness	-0.022	0.261	-0.004	0.862	-0.025	0.183	-0.009	0.650	0.017	0.519	-0.008	0.731
S_orbital.H_Shaped_thickness	-0.025	0.188	-0.042	0.076	-0.031	0.104	-0.013	0.527	-0.026	0.266	-0.025	0.183
S_suborbital_thickness	0.031	0.112	0.040	0.104	0.005	0.799	0.011	0.598	0.026	0.303	-0.003	0.874
Lat_Fis.ant.Horizont_thickness	-0.004	0.830	0.006	0.808	0.016	0.458	-0.019	0.327	-0.027	0.288	-0.026	0.173
Lat_Fis.ant.Vertical_thickness	0.004	0.830	0.017	0.519	0.007	0.786	-0.021	0.279	-0.028	0.266	-0.044	0.018
Lat_Fis.post_thickness	-0.036	0.063	-0.074	0.001	-0.048	0.010	-0.064	0.001	-0.073	0.001	-0.078	0.000
S_pericallosal_thickness	0.004	0.830	-0.015	0.576	-0.055	0.004	0.031	0.117	0.015	0.571	-0.015	0.483
Amygdala	-0.070	0.000	-0.103	0.000	-0.062	0.001	-0.060	0.000	-0.112	0.000	-0.052	0.005
Hippocampus	-0.085	0.000	-0.120	0.000	-0.097	0.000	-0.103	0.000	-0.132	0.000	-0.088	0.000

Table S5. Associations between discrimination and cortical thickness.

	Left Hemisphere						*Right Hemisphere*					
Region	*GLM (Age + Sex)*		*GLM (Age + Sex + Site*		*Brainchart Deviatoins*		*GLM (Age + Sex)*		*GLM (Age + Sex + Site*		*Brainchart Deviatoins*	
	Estimate (β)	FDR (p)	Estimate (β)	FDR (p)	Estimate (β)	FDR (p)	Estimate (β)	FDR (p)	Estimate (β)	FDR (p)	Estimate (β)	FDR (p)
G.S_frontomargin_thickness	-0.008	0.781	-0.001	0.971	-0.003	0.928	-0.028	0.205	-0.023	0.344	-0.023	0.404
G.S_transv_frontopol_thickness	-0.001	0.983	0.005	0.878	0.005	0.920	0.008	0.781	0.012	0.783	0.014	0.709
G.S_cingul.Ant_thickness	-0.012	0.725	-0.008	0.872	-0.006	0.920	-0.010	0.780	-0.005	0.872	-0.002	0.955
G.S_cingul.Mid.Ant_thickness	-0.015	0.613	-0.013	0.738	-0.013	0.738	-0.017	0.552	-0.011	0.783	-0.014	0.712
G.S_cingul.Mid.Post_thickness	0.001	0.973	0.003	0.916	0.001	0.963	0.003	0.932	0.006	0.872	0.004	0.920
G_front_inf.Opercular_thickness	0.014	0.633	0.014	0.738	0.019	0.471	-0.002	0.973	-0.006	0.872	0.000	0.974
G_front_inf.Orbital_thickness	-0.042	0.051	-0.038	0.075	-0.038	0.114	-0.008	0.781	-0.006	0.872	-0.004	0.927
G_front_inf.Triangul_thickness	-0.034	0.122	-0.032	0.136	-0.030	0.238	-0.011	0.754	-0.012	0.783	-0.005	0.920
G_front_middle_thickness	-0.006	0.828	-0.005	0.872	-0.001	0.963	0.002	0.973	-0.001	0.964	0.004	0.920
G_front_sup_thickness	0.000	0.986	0.000	0.998	0.006	0.920	-0.001	0.973	-0.001	0.964	0.003	0.927
G_Ins_lg.S_cent_ins_thickness	0.003	0.932	0.006	0.872	0.006	0.920	0.005	0.886	0.005	0.872	0.004	0.920
G_insular_short_thickness	-0.006	0.828	-0.004	0.884	-0.004	0.920	0.006	0.828	0.007	0.872	0.008	0.920
G_orbital_thickness	-0.030	0.195	-0.026	0.287	-0.025	0.383	-0.028	0.205	-0.024	0.335	-0.022	0.405
G_rectus_thickness	-0.001	0.983	0.005	0.872	0.006	0.920	-0.009	0.781	-0.005	0.872	-0.007	0.920
G_subcallosal_thickness	0.015	0.628	0.011	0.783	0.017	0.603	0.027	0.219	0.027	0.256	0.026	0.372
S_circular_insula_ant_thickness	-0.019	0.454	-0.015	0.714	-0.016	0.680	-0.023	0.290	-0.018	0.539	-0.022	0.405
S_circular_insula_inf_thickness	-0.013	0.725	-0.009	0.872	-0.007	0.920	-0.021	0.375	-0.019	0.500	-0.014	0.709
S_circular_insula_sup_thickness	-0.039	0.051	-0.036	0.075	-0.036	0.114	-0.037	0.078	-0.036	0.075	-0.036	0.114
S_front_inf_thickness	-0.017	0.528	-0.013	0.738	-0.011	0.846	-0.012	0.725	-0.010	0.862	-0.008	0.920
S_front_middle_thickness	-0.025	0.233	-0.022	0.361	-0.019	0.471	-0.008	0.781	-0.002	0.947	-0.006	0.920
S_front_sup_thickness	-0.004	0.912	-0.002	0.947	-0.002	0.957	0.006	0.828	0.009	0.862	0.007	0.920
S_orbital_lateral_thickness	-0.025	0.233	-0.019	0.525	-0.023	0.404	-0.012	0.725	-0.009	0.862	-0.008	0.920
S_orbital_med.olfact_thickness	0.032	0.177	0.034	0.110	0.034	0.123	0.020	0.443	0.022	0.385	0.020	0.471
S_orbital.H_Shaped_thickness	-0.010	0.780	-0.004	0.891	-0.006	0.920	-0.031	0.184	-0.030	0.136	-0.031	0.214
S_suborbital_thickness	-0.008	0.781	-0.004	0.884	-0.001	0.963	-0.006	0.828	-0.005	0.872	-0.003	0.928
Lat_Fis.ant.Horizont_thickness	-0.026	0.233	-0.023	0.361	-0.025	0.383	-0.011	0.739	-0.010	0.862	-0.009	0.920
Lat_Fis.ant.Vertical_thickness	-0.011	0.760	-0.008	0.872	-0.005	0.920	-0.019	0.454	-0.017	0.629	-0.014	0.709
Lat_Fis.post_thickness	-0.041	0.051	-0.037	0.075	-0.035	0.123	-0.027	0.219	-0.026	0.256	-0.023	0.404
S_pericallosal_thickness	-0.009	0.781	-0.008	0.872	-0.008	0.920	0.015	0.628	0.014	0.738	0.014	0.712
Amygdala	-0.044	0.022	-0.042	0.017	-0.042	0.077	-0.049	0.010	-0.045	0.017	-0.045	0.071

Hippocampus	-0.024	0.233	-0.025	0.256	-0.020	0.471	-0.027	0.201	-0.029	0.154	-0.024	0.404

Table S6. Associations between state-level racism and cortical thickness.

	Left Hemisphere						*Right Hemisphere*					
Region	*GLM (Age + Sex)*		*GLM (Age + Sex + Site*		*Brainchart Deviatoins*		*GLM (Age + Sex)*		*GLM (Age + Sex + Site*		*Brainchart Deviatoins*	
	Estimate (β)	FDR (p)	Estimate (β)	FDR (p)	Estimate (β)	FDR (p)	Estimate (β)	FDR (p)	Estimate (β)	FDR (p)	Estimate (β)	FDR (p)
G.S_frontomargin_thickness	-0.026	0.136	-0.052	0.999	-0.017	0.286	-0.067	0.000	-0.178	0.892	0.022	0.159
G.S_transv_frontopol_thickness	-0.092	0.000	0.054	0.999	0.004	0.808	-0.137	0.000	-0.137	0.939	-0.010	0.536
G.S_cingul.Ant_thickness	-0.006	0.743	0.240	0.833	0.089	0.000	-0.030	0.071	0.191	0.892	0.094	0.000
G.S_cingul.Mid.Ant_thickness	-0.051	0.002	-0.002	0.999	0.070	0.000	0.018	0.284	-0.230	0.833	0.122	0.000
G.S_cingul.Mid.Post_thickness	-0.056	0.000	-0.025	0.999	0.079	0.000	0.020	0.235	0.085	0.999	0.094	0.000
G_front_inf.Opercular_thickness	0.027	0.114	0.148	0.939	-0.010	0.536	0.015	0.387	-0.006	0.999	0.003	0.874
G_front_inf.Orbital_thickness	-0.008	0.660	-0.152	0.939	0.001	0.951	-0.022	0.201	-0.167	0.939	-0.015	0.348
G_front_inf.Triangul_thickness	-0.022	0.201	-0.180	0.892	-0.058	0.000	-0.067	0.000	-0.229	0.833	-0.044	0.003
G_front_middle_thickness	-0.033	0.049	0.014	0.999	-0.067	0.000	-0.072	0.000	-0.026	0.999	-0.069	0.000
G_front_sup_thickness	-0.063	0.000	-0.040	0.999	0.051	0.001	-0.053	0.001	-0.005	0.999	0.049	0.001
G_Ins_lg.S_cent_ins_thickness	0.046	0.005	-0.094	0.999	0.069	0.000	0.005	0.780	-0.069	0.999	0.058	0.000
G_insular_short_thickness	0.024	0.160	0.017	0.999	0.109	0.000	0.026	0.136	0.139	0.939	0.086	0.000
G_orbital_thickness	0.022	0.201	0.032	0.999	0.111	0.000	0.010	0.566	-0.235	0.833	0.092	0.000
G_rectus_thickness	0.074	0.000	-0.090	0.999	0.149	0.000	0.011	0.566	-0.006	0.999	0.132	0.000
G_subcallosal_thickness	0.083	0.000	-0.051	0.999	-0.076	0.000	0.065	0.000	-0.197	0.892	-0.087	0.000
S_circular_insula_ant_thickness	-0.006	0.743	-0.306	0.833	0.086	0.000	-0.003	0.835	-0.156	0.939	0.098	0.000
S_circular_insula_inf_thickness	0.030	0.071	-0.254	0.833	0.052	0.001	-0.035	0.038	-0.283	0.833	0.042	0.005
S_circular_insula_sup_thickness	0.017	0.299	0.099	0.999	0.117	0.000	-0.062	0.000	-0.039	0.999	0.160	0.000
S_front_inf_thickness	0.056	0.000	-0.018	0.999	-0.131	0.000	-0.049	0.002	0.062	0.999	-0.095	0.000
S_front_middle_thickness	0.020	0.235	0.174	0.892	-0.098	0.000	-0.117	0.000	0.096	0.999	-0.081	0.000
S_front_sup_thickness	0.069	0.000	0.062	0.999	-0.064	0.000	-0.001	0.919	-0.068	0.999	-0.016	0.304
S_orbital_lateral_thickness	0.033	0.052	-0.130	0.939	-0.090	0.000	-0.059	0.000	-0.236	0.833	-0.109	0.000
S_orbital_med.olfact_thickness	0.013	0.464	0.051	0.999	0.143	0.000	-0.023	0.181	-0.191	0.892	0.062	0.000
S_orbital.H_Shaped_thickness	-0.044	0.007	0.215	0.862	0.090	0.000	-0.024	0.160	-0.117	0.953	0.127	0.000
S_suborbital_thickness	-0.061	0.000	-0.131	0.939	0.031	0.047	-0.050	0.002	0.109	0.999	0.074	0.000
Lat_Fis.ant.Horizont_thickness	-0.013	0.466	0.046	0.999	-0.052	0.001	-0.060	0.000	0.023	0.999	-0.016	0.304
Lat_Fis.ant.Vertical_thickness	0.012	0.518	-0.084	0.999	-0.073	0.000	-0.040	0.016	-0.153	0.939	-0.025	0.111
Lat_Fis.post_thickness	0.043	0.008	0.080	0.999	0.103	0.000	0.008	0.660	-0.266	0.833	0.098	0.000
S_pericallosal_thickness	0.010	0.584	0.020	0.999	0.066	0.000	0.049	0.002	0.000	0.999	0.008	0.617
Amygdala	-0.058	0.000	-0.245	0.833	0.133	0.000	-0.045	0.002	-0.465	0.097	0.125	0.000
Hippocampus	-0.009	0.584	-0.460	0.097	0.102	0.000	-0.003	0.835	-0.419	0.151	0.090	0.000

Table S7. Within-group associations between income and cortical thickness (Z-deviations).

	Left Hemisphere				Right Hemisphere			
Region	*White individuals*		*Black individuals*		*White individuals*		*Black individuals*	
	Estimate (*b*)	FDR (p)	Estimate (*b*)	FDR (p)	Estimate (*b*)	FDR (p)	Estimate (*b*)	FDR (p)
G.S_frontomargin_thickness	0.009	0.456	0.029	0.544	0.001	0.985	0.005	0.906
G.S_transv_frontopol_thickness	0.001	0.985	0.020	0.544	0.003	0.876	0.004	0.906
G.S_cingul.Ant_thickness	-0.002	0.911	-0.016	0.544	0.006	0.528	-0.001	0.960
G.S_cingul.Mid.Ant_thickness	0.005	0.456	-0.001	0.960	0.008	0.327	0.014	0.544
G.S_cingul.Mid.Post_thickness	0.023	0.006	-0.024	0.544	0.021	0.007	-0.006	0.892
G_front_inf.Opercular_thickness	0.013	0.161	0.004	0.906	0.017	0.055	0.006	0.906
G_front_inf.Orbital_thickness	0.016	0.084	0.001	0.960	-0.002	0.914	-0.015	0.604
G_front_inf.Triangul_thickness	0.000	0.985	-0.006	0.906	0.003	0.876	-0.016	0.604
G_front_middle_thickness	-0.008	0.549	-0.008	0.892	0.000	0.994	-0.019	0.544
G_front_sup_thickness	0.004	0.714	0.008	0.840	0.007	0.577	0.004	0.906
G_Ins_lg.S_cent_ins_thickness	0.006	0.598	0.005	0.906	0.009	0.456	0.018	0.544
G_insular_short_thickness	0.014	0.150	0.017	0.544	0.011	0.315	0.009	0.840
G_orbital_thickness	0.016	0.128	0.023	0.544	0.009	0.393	0.008	0.840
G_rectus_thickness	-0.007	0.456	0.016	0.544	-0.007	0.456	0.003	0.906
G_subcallosal_thickness	-0.004	0.714	0.006	0.892	0.000	0.985	0.012	0.544
S_circular_insula_ant_thickness	0.009	0.412	0.005	0.906	0.003	0.855	0.003	0.906
S_circular_insula_inf_thickness	0.019	0.014	0.028	0.544	0.015	0.094	0.017	0.544
S_circular_insula_sup_thickness	0.026	0.001	0.014	0.544	0.021	0.010	0.009	0.840
S_front_inf_thickness	0.013	0.209	0.001	0.960	0.000	0.994	-0.003	0.906
S_front_middle_thickness	-0.001	0.985	-0.014	0.604	0.001	0.981	-0.019	0.544
S_front_sup_thickness	0.008	0.428	0.013	0.637	-0.002	0.876	-0.009	0.840
S_orbital_lateral_thickness	-0.001	0.985	-0.009	0.840	0.009	0.456	-0.005	0.906
S_orbital_med.olfact_thickness	-0.012	0.398	0.016	0.655	-0.009	0.456	0.023	0.544
S_orbital.H_Shaped_thickness	0.004	0.733	0.013	0.669	0.012	0.240	-0.003	0.906
S_suborbital_thickness	-0.008	0.456	-0.022	0.544	0.006	0.598	-0.003	0.906
Lat_Fis.ant.Horizont_thickness	0.014	0.161	0.011	0.759	0.009	0.398	0.004	0.906
Lat_Fis.ant.Vertical_thickness	0.008	0.456	0.009	0.840	-0.004	0.801	-0.020	0.544
Lat_Fis.post_thickness	0.026	0.002	0.003	0.906	0.023	0.007	-0.007	0.892
S_pericallosal_thickness	-0.012	0.062	0.000	0.975	-0.008	0.295	-0.013	0.544
Amygdala	0.049	0.000	0.030	0.544	0.034	0.001	0.025	0.544
Hippocampus	0.050	0.000	0.030	0.544	0.048	0.000	0.014	0.743

Table S8. Within-group associations between ADI and cortical thickness (Z-deviations).

	Left Hemisphere				Right Hemisphere			
Region	*White individuals*		*Black individuals*		*White individuals*		*Black individuals*	
	Estimate (*b*)	FDR (p)	Estimate (*b*)	FDR (p)	Estimate (*b*)	FDR (p)	Estimate (*b*)	FDR (p)
G.S_frontomargin_thickness	-0.001	0.625	-0.002	0.479	-0.001	0.079	-0.003	0.075
G.S_transv_frontopol_thickness	0.000	0.984	0.000	0.983	-0.002	0.038	-0.002	0.389
G.S_cingul.Ant_thickness	-0.001	0.109	-0.004	0.047	-0.002	0.027	-0.003	0.089
G.S_cingul.Mid.Ant_thickness	-0.001	0.048	-0.002	0.070	-0.001	0.110	-0.003	0.047
G.S_cingul.Mid.Post_thickness	0.000	0.964	-0.002	0.179	0.000	0.585	-0.003	0.075
G_front_inf.Opercular_thicknes s	0.000	0.842	-0.001	0.563	-0.001	0.495	-0.002	0.208
G_front_inf.Orbital_thickness	0.000	0.779	-0.002	0.471	-0.001	0.426	-0.003	0.151
G_front_inf.Triangul_thickness	0.000	0.701	-0.001	0.807	0.000	0.959	-0.003	0.190
G_front_middle_thickness	-0.001	0.249	-0.004	0.075	-0.001	0.326	-0.005	0.047
G_front_sup_thickness	-0.001	0.074	-0.002	0.207	-0.001	0.110	-0.004	0.085
G_Ins_lg.S_cent_ins_thickness	0.001	0.625	0.000	0.910	0.000	0.701	-0.001	0.534
G_insular_short_thickness	0.001	0.284	0.000	0.910	0.000	0.715	-0.002	0.389
G_orbital_thickness	0.000	0.967	-0.002	0.389	-0.001	0.048	-0.002	0.300
G_rectus_thickness	-0.001	0.495	0.000	0.906	-0.002	0.027	-0.002	0.260
G_subcallosal_thickness	-0.002	0.040	0.000	0.910	-0.001	0.040	-0.002	0.189
S_circular_insula_ant_thickness	-0.001	0.489	0.000	0.910	-0.001	0.457	0.000	0.910
S_circular_insula_inf_thickness	0.000	0.574	-0.001	0.519	0.000	0.833	0.001	0.701
S_circular_insula_sup_thickness	0.000	0.715	-0.001	0.745	0.000	0.743	-0.002	0.190
S_front_inf_thickness	-0.001	0.487	-0.002	0.389	-0.001	0.284	-0.004	0.057
S_front_middle_thickness	0.000	0.927	-0.005	0.003	-0.001	0.426	-0.006	0.001
S_front_sup_thickness	0.000	0.625	-0.002	0.190	-0.001	0.114	-0.003	0.075
S_orbital_lateral_thickness	0.001	0.495	0.001	0.469	0.000	0.959	-0.003	0.125
S_orbital_med.olfact_thickness	0.000	0.715	-0.002	0.514	-0.001	0.533	-0.001	0.563
S_orbital.H_Shaped_thickness	-0.001	0.207	0.000	0.969	0.000	0.927	-0.001	0.647
S_suborbital_thickness	0.000	0.701	-0.001	0.471	0.000	0.973	-0.001	0.638
Lat_Fis.ant.Horizont_thickness	0.001	0.228	0.000	0.949	-0.001	0.426	-0.001	0.563
Lat_Fis.ant.Vertical_thickness	0.000	0.960	-0.001	0.668	-0.002	0.050	-0.003	0.207
Lat_Fis.post_thickness	0.000	0.996	-0.001	0.563	-0.001	0.180	-0.001	0.563
S_pericallosal_thickness	-0.001	0.040	-0.001	0.483	0.000	0.536	0.000	0.949
Amygdala	-0.001	0.207	0.001	0.563	-0.001	0.495	0.001	0.745
Hippocampus	-0.003	0.026	0.000	0.917	-0.002	0.027	0.001	0.595

Table S9. Within-group associations between discrimination and cortical thickness (Z-deviations).

	Left Hemisphere				Right Hemisphere			
Region	*White individuals*		*Black individuals*		*White individuals*		*Black individuals*	
	Estimate (*b*)	FDR (p)	Estimate (*b*)	FDR (p)	Estimate (*b*)	FDR (p)	Estimate (*b*)	FDR (p)
G.S_frontomargin_thickness	0.007	0.769	-0.008	0.570	0.001	0.906	-0.013	0.218
G.S_transv_frontopol_thickness	0.006	0.769	-0.006	0.690	0.004	0.769	-0.001	0.957
G.S_cingul.Ant_thickness	0.004	0.769	-0.013	0.195	0.005	0.769	-0.011	0.330
G.S_cingul.Mid.Ant_thickness	0.005	0.728	-0.011	0.178	0.000	0.973	-0.002	0.798
G.S_cingul.Mid.Post_thickness	0.007	0.728	-0.002	0.798	0.009	0.728	-0.007	0.497
G_front_inf.Opercular_thicknes s	0.005	0.769	-0.004	0.740	0.002	0.864	-0.014	0.195
G_front_inf.Orbital_thickness	-0.002	0.857	-0.020	0.178	0.005	0.769	-0.011	0.338
G_front_inf.Triangul_thickness	-0.002	0.857	-0.018	0.178	0.004	0.769	-0.012	0.330
G_front_middle_thickness	0.003	0.857	-0.009	0.552	0.003	0.857	-0.005	0.740
G_front_sup_thickness	0.003	0.857	-0.004	0.740	0.004	0.772	-0.007	0.690
G_Ins_lg.S_cent_ins_thickness	0.002	0.864	0.002	0.822	-0.002	0.864	0.003	0.795
G_insular_short_thickness	0.002	0.857	-0.005	0.740	0.004	0.769	-0.004	0.740
G_orbital_thickness	0.002	0.857	-0.017	0.195	0.004	0.769	-0.017	0.178
G_rectus_thickness	0.007	0.728	-0.006	0.603	0.003	0.857	-0.009	0.407
G_subcallosal_thickness	0.004	0.769	0.003	0.740	0.007	0.728	0.003	0.740
S_circular_insula_ant_thickness	0.004	0.769	-0.005	0.701	0.003	0.857	-0.008	0.466
S_circular_insula_inf_thickness	0.002	0.857	-0.008	0.470	-0.003	0.857	-0.001	0.913
S_circular_insula_sup_thickness	-0.001	0.906	-0.012	0.207	-0.001	0.893	-0.014	0.195
S_front_inf_thickness	0.006	0.769	-0.013	0.199	0.005	0.769	-0.013	0.276
S_front_middle_thickness	0.001	0.904	-0.010	0.330	0.002	0.857	-0.003	0.795
S_front_sup_thickness	0.001	0.917	0.001	0.913	0.004	0.769	0.000	0.966
S_orbital_lateral_thickness	-0.002	0.857	-0.004	0.740	0.002	0.864	-0.003	0.795
S_orbital_med.olfact_thickness	0.010	0.728	0.017	0.195	0.011	0.728	-0.010	0.496
S_orbital.H_Shaped_thickness	0.004	0.769	-0.011	0.301	-0.002	0.864	-0.014	0.195
S_suborbital_thickness	-0.002	0.861	0.001	0.913	0.002	0.857	-0.009	0.496
Lat_Fis.ant.Horizont_thickness	-0.006	0.769	-0.003	0.798	0.003	0.857	-0.013	0.218
Lat_Fis.ant.Vertical_thickness	0.004	0.769	-0.016	0.178	-0.006	0.769	-0.005	0.740
Lat_Fis.post_thickness	-0.003	0.857	-0.006	0.701	0.000	0.973	-0.003	0.797
S_pericallosal_thickness	0.000	0.973	-0.004	0.644	0.005	0.728	-0.004	0.705
Amygdala	-0.008	0.769	-0.007	0.701	-0.009	0.728	-0.007	0.644
Hippocampus	0.000	0.973	0.003	0.822	0.001	0.973	-0.003	0.822

Table S10. Within-group associations between state-level racism and cortical thickness (Z-deviations).

	Left Hemisphere				Right Hemisphere			
Region	*White individuals*		*Black individuals*		*White individuals*		*Black individuals*	
	Estimate (*b*)	FDR (p)	Estimate (*b*)	FDR (p)	Estimate (*b*)	FDR (p)	Estimate (*b*)	FDR (p)
G.S_frontomargin_thickness	-0.024	0.159	0.030	0.816	0.022	0.145	0.008	0.929
G.S_transv_frontopol_thickness	-0.002	0.946	0.049	0.651	-0.012	0.456	0.004	0.937
G.S_cingul.Ant_thickness	0.078	0.000	0.135	0.042	0.082	0.000	0.139	0.042
G.S_cingul.Mid.Ant_thickness	0.051	0.000	0.027	0.692	0.092	0.000	0.090	0.061
G.S_cingul.Mid.Post_thickness	0.087	0.000	-0.011	0.929	0.085	0.000	0.090	0.134
G_front_inf.Opercular_thickness	-0.015	0.333	0.033	0.761	0.000	0.986	0.019	0.857
G_front_inf.Orbital_thickness	0.001	0.964	0.007	0.929	-0.015	0.373	-0.017	0.874
G_front_inf.Triangul_thickness	-0.061	0.000	-0.026	0.818	-0.053	0.001	0.010	0.929
G_front_middle_thickness	-0.099	0.000	0.099	0.261	-0.098	0.000	0.054	0.651
G_front_sup_thickness	0.053	0.000	0.005	0.935	0.054	0.002	0.048	0.676
G_Ins_lg.S_cent_ins_thickness	0.078	0.000	0.044	0.663	0.058	0.001	0.091	0.261
G_insular_short_thickness	0.108	0.000	0.115	0.084	0.077	0.000	0.180	0.005
G_orbital_thickness	0.119	0.000	0.158	0.050	0.085	0.000	0.084	0.261
G_rectus_thickness	0.132	0.000	0.099	0.094	0.115	0.000	0.131	0.042
G_subcallosal_thickness	-0.078	0.000	0.015	0.857	-0.084	0.000	0.024	0.756
S_circular_insula_ant_thickness	0.098	0.000	-0.068	0.356	0.105	0.000	0.019	0.843
S_circular_insula_inf_thickness	0.044	0.001	0.060	0.453	0.041	0.005	0.024	0.818
S_circular_insula_sup_thickness	0.116	0.000	0.054	0.498	0.160	0.000	0.117	0.061
S_front_inf_thickness	-0.141	0.000	-0.027	0.816	-0.116	0.000	0.088	0.276
S_front_middle_thickness	-0.115	0.000	0.060	0.498	-0.107	0.000	0.134	0.056
S_front_sup_thickness	-0.071	0.000	0.027	0.816	-0.034	0.029	0.148	0.042
S_orbital_lateral_thickness	-0.085	0.000	-0.054	0.517	-0.129	0.000	-0.042	0.726
S_orbital_med.olfact_thickness	0.178	0.000	0.156	0.056	0.064	0.000	0.108	0.237
S_orbital.H_Shaped_thickness	0.086	0.000	0.069	0.387	0.130	0.000	0.055	0.531
S_suborbital_thickness	0.033	0.030	-0.007	0.929	0.082	0.000	0.027	0.816
Lat_Fis.ant.Horizont_thickness	-0.052	0.001	-0.050	0.562	-0.027	0.085	0.084	0.296
Lat_Fis.ant.Vertical_thickness	-0.071	0.000	-0.044	0.657	-0.031	0.073	0.012	0.929
Lat_Fis.post_thickness	0.109	0.000	0.060	0.499	0.115	0.000	-0.026	0.816
S_pericallosal_thickness	0.051	0.000	-0.006	0.929	0.013	0.250	-0.059	0.261
Amygdala	0.194	0.000	-0.025	0.843	0.173	0.000	-0.062	0.531
Hippocampus	0.148	0.000	-0.007	0.935	0.137	0.000	-0.042	0.756

Table S11. Parallel mediation of race-related variability by racial inequities using Z-score deviations.

	Left Hemisphere							*Right Hemisphere*						
Region	**Total Effect ©**	**p**	**Total Indirect Effect (ab)**	**p**	**Direct Effect (c')**	**p**	**Percentage mediated (%)**	**Total Effect ©**	**p**	**Total Indirect Effect (ab)**	**p**	**Direct Effect (c')**	**p**	**Percentage mediated (%)**
G.S_frontomargin_thickness	0.082	<0.001	0.026	0.002	0.108	<0.001	24.271	0.122	<0.001	0.027	0.002	0.149	<0.001	17.836
G.S_transv_frontopol_thickness	0.092	<0.001	0.029	0.001	0.121	<0.001	23.668	0.035	0.038	0.026	0.004	0.061	<0.001	41.967
G.S_cingul.Ant_thickness	0.141	<0.001	0.039	<0.001	0.18	<0.001	21.538	0.023	0.193	0.033	<0.001	0.056	<0.001	.
G.S_cingul.Mid.Ant_thickness	0.042	0.011	0.029	0.001	0.071	<0.001	40.992	0.056	0.001	-0.022	0.009	0.034	0.018	-65.145
G.S_cingul.Mid.Post_thickness	0.013	0.446	0.005	0.618	0.018	0.228	.	0.005	0.726	0.024	0.006	0.029	0.028	.
G_front_inf.Opercular_thickness	-0.007	0.677	0.004	0.654	-0.003	0.836	.	0.075	<0.001	0.001	0.901	0.076	<0.001	.
G_front_inf.Orbital_thickness	0.078	<0.001	0.022	0.009	0.101	<0.001	22.231	-0.053	0.001	0.034	<0.001	-0.02	0.157	-171.791
G_front_inf.Triangul_thickness	0.098	<0.001	0.007	0.45	0.105	<0.001	.	0.003	0.845	0.027	0.002	0.03	0.039	.
G_front_middle_thickness	0.091	<0.001	-0.009	0.312	0.082	<0.001	.	0.054	0.001	0.034	<0.001	0.088	<0.001	38.145
G_front_sup_thickness	0.094	<0.001	-0.02	0.021	0.074	<0.001	-26.69	0.043	0.009	0.028	0.002	0.071	<0.001	39.064
G_Ins_lg.S_cent_ins_thickness	0.141	<0.001	0.036	<0.001	0.178	<0.001	20.507	0.056	0.001	0.017	0.062	0.074	<0.001	.
G_insular_short_thickness	-0.095	<0.001	0.02	0.028	-0.076	<0.001	-25.774	0.042	0.01	0.018	0.032	0.06	<0.001	30.529
G_orbital_thickness	0.044	0.01	0.03	0.001	0.074	<0.001	39.965	-0.053	0.003	0.028	0.002	-0.024	0.103	-114.602
G_rectus_thickness	0.008	0.657	0.01	0.276	0.018	0.228	.	0.148	<0.001	0.015	0.091	0.163	<0.001	.
G_subcallosal_thickness	-0.011	0.498	0.023	0.013	0.011	0.424	.	0.037	0.03	0.021	0.017	0.058	<0.001	36.044
S_circular_insula_ant_thickness	-0.029	0.094	0.021	0.024	-0.008	0.58	.	0.033	0.053	-0.001	0.868	0.031	0.031	.
S_circular_insula_inf_thickness	0.007	0.679	-0.01	0.228	-0.004	0.784	.	0.231	<0.001	0.042	<0.001	0.273	<0.001	15.261
S_circular_insula_sup_thickness	0.033	0.042	-0.011	0.199	0.022	0.12	.	0.143	<0.001	0.042	<0.001	0.184	<0.001	22.585
S_front_inf_thickness	0.066	<0.001	0.031	0.001	0.097	<0.001	31.826	0.101	<0.001	0.048	<0.001	0.149	<0.001	32.309
S_front_middle_thickness	0.08	<0.001	0.021	0.017	0.101	<0.001	20.721	0.143	<0.001	0.003	0.729	0.146	<0.001	.
S_front_sup_thickness	0.16	<0.001	0.008	0.386	0.168	<0.001	.	0.011	0.511	0.032	<0.001	0.043	0.003	.
S_orbital_lateral_thickness	0.188	<0.001	0.027	0.001	0.215	<0.001	12.524	0.129	<0.001	0.022	0.01	0.151	<0.001	14.786
S_orbital_med.olfact_thickness	0.108	<0.001	0.005	0.557	0.113	<0.001	.	0.117	<0.001	-0.017	0.055	0.1	<0.001	.
S_orbital.H_Shaped_thickness	0.081	<0.001	0.009	0.335	0.09	<0.001	.	0.076	<0.001	0.03	0.001	0.106	<0.001	28.017
S_suborbital_thickness	0.016	0.349	0.015	0.097	0.031	0.035	.	0.066	<0.001	0.046	<0.001	0.112	<0.001	40.979

Lat_Fis.ant.Horizont_thickness	0.142	<0.001	0.026	0.002	0.168	<0.001	15.285	0.046	0.003	0.015	0.077	0.061	<0.001	.
Lat_Fis.ant.Vertical_thickness	0.133	<0.001	0.023	0.006	0.157	<0.001	14.931	0.127	<0.001	0.037	<0.001	0.164	<0.001	22.688
Lat_Fis.post_thickness	0.123	<0.001	0.006	0.437	0.13	<0.001	.	0.165	<0.001	0.031	<0.001	0.195	<0.001	15.736
S_pericallosal_thickness	0.208	<0.001	0.016	0.055	0.225	<0.001	.	0.085	<0.001	0.002	0.808	0.087	<0.001	.
Amygdala	0.012	0.482	0.026	0.004	0.038	0.006	.	0.073	<0.001	0.027	0.001	0.101	<0.001	27.133
Hippocampus	0.044	0.008	0.023	0.01	0.067	<0.001	34.746	0.048	0.005	0.035	<0.001	0.083	<0.001	41.804

Note: Brain region labels are short name (G = Gyrus, S = Sulcus, Lat = Lateral, Med = Medial). Percent mediated is presented only for regions which showed partial or full mediation (i.e., significant total and indirect effect).

Supplementary Note.

Equations for linear models

$Y_{thickness} = X_{Age} + X_{Sex} + X_{Race} + e$

$Y_{thickness} = X_{Age} + X_{Sex} + X_{Race} + X_{site} + e$

$Y_{Z\text{-Deivation Thickness}} = X_{Race} + e$

$Y_{thickness} = X_{Age} + X_{Sex} + X_{Income} + X_{ADI} + X_{Discrimination} + X_{State\text{-Level Racism}} + e$

$Y_{thickness} = X_{Age} + X_{Sex} + X_{Income} + X_{ADI} + X_{Discrimination} + X_{State\text{-Level Racism}} + X_{site} + e$

$Y_{Z\text{-Deivation Thickness}} = X_{Income} + X_{ADI} + X_{Discrimination} + X_{State\text{-Level Racism}} + e$
